# Socially guided individual learning mitigates the double-edged sword of pedagogy

**DOI:** 10.1098/rspb.2026.0619

**Published:** 2026-10-14

**Authors:** Jérémy Perez, Maxime Derex

**Affiliations:** ^1^ Flowers AI & CogSci Lab, Inria Centre de recherche Bordeaux Sud-Ouest, Talence, France; ^2^ IAST, Toulouse, Occitanie, France

**Keywords:** cultural evolution, social learning, innovation, cumulative culture, heuristics, exploration, transmission chain

## Abstract

Social learning and innovation are fundamental processes in cultural evolution, yet their interaction remains poorly understood. Prior research suggests that inheriting social information reduces exploration. However, most studies have focused on the transmission of fully fledged solutions. Here, we investigate whether alternative types of information can facilitate cumulative cultural evolution without reducing exploration. Specifically, we conducted a pre-registered experiment with 400 adult participants comparing the inheritance of solutions with that of heuristics—simple rules that guide problem solving. We found that social learners who inherited a heuristic explored more frequently and broadly than those who received a solution. Their productions were also less similar to those of their demonstrators, suggesting that heuristics foster more independent exploration. Despite these differences, both heuristics and solutions enabled learners to surpass their demonstrator's performance. These findings highlight that the type of social information transmitted plays a crucial role in the evolvability of culture.

## 1. Introduction

The capacity to build on the knowledge gathered by previous generations has been argued to be uniquely human [1–3], and is considered a key factor in the ecological success of the human species [4]. The two key ingredients of cumulative cultural evolution (CCE) are social learning and innovation [5,6]. While social learning is fundamental to ensure that the insights gathered by a given generation are transmitted to the next, innovation is just as crucial to allow cultural groups to refine their cultural repertoire over time and adapt to new environments.

Despite their importance, the interplay between these two processes remains poorly understood. Existing research suggests the existence of a trade-off between relying on social information and innovating. Indeed, knowledge about a previously discovered solution appears to constrain future exploration, ultimately preventing learners from discovering more efficient solutions [7]. This phenomenon has been repeatedly observed both theoretically and empirically by researchers in diverse disciplines, such as developmental psychology [8], design studies [9] and cultural evolution [7,10,11], in both adults and children. As a consequence, it has been argued that a ‘challenge for educators may be how to foster learners capable of doing new things while simultaneously teaching what other generations have done’ [8, p. 11].

However, all of these previous works have focused on a very specific way of transmitting social information; namely, communicating information about a previously discovered *solution*. This narrow focus is in contrast with the wide diversity of pathways used by humans to transmit accumulated knowledge. Ethnographic research indicates that cultural demonstrators often provide learners with guiding principles rather than specific solutions. Finnish mushroom foragers, for instance, use such principles to guide their decisions, such as choosing between foraging locations (‘the older the forest, the more mushrooms’) and identifying edible mushrooms (‘avoid white mushrooms altogether’) [12]. Similar heuristics are also used in the production of technological artefacts [13]. For instance, rules of thumb guide the dimensions of Marshallese canoes (e.g. ‘the length of the canoe is equal to the length of the mast’ [14]), bows (e.g. ‘a good bow was one whose string made a high musical note when tapped with an arrow or snapped with the fingers’ [15, p. 110]) and Viking ships [16].

Compared with solutions, heuristics do not provide learners with precise knowledge of the best-known solution. Instead, they specify constraints that, when followed, can lead to the creation of an efficient solution. These two types of cultural information are likely to have contrasting effects on innovation. While solutions provide learners with a specific point in the design space known to yield high pay-offs, they reveal little about alternative solutions that may offer equally good or even superior results. By contrast, heuristics do not offer a fixed starting point for exploration but rather serve as guidelines for navigating the design space (figure 1).

**Figure 1.**
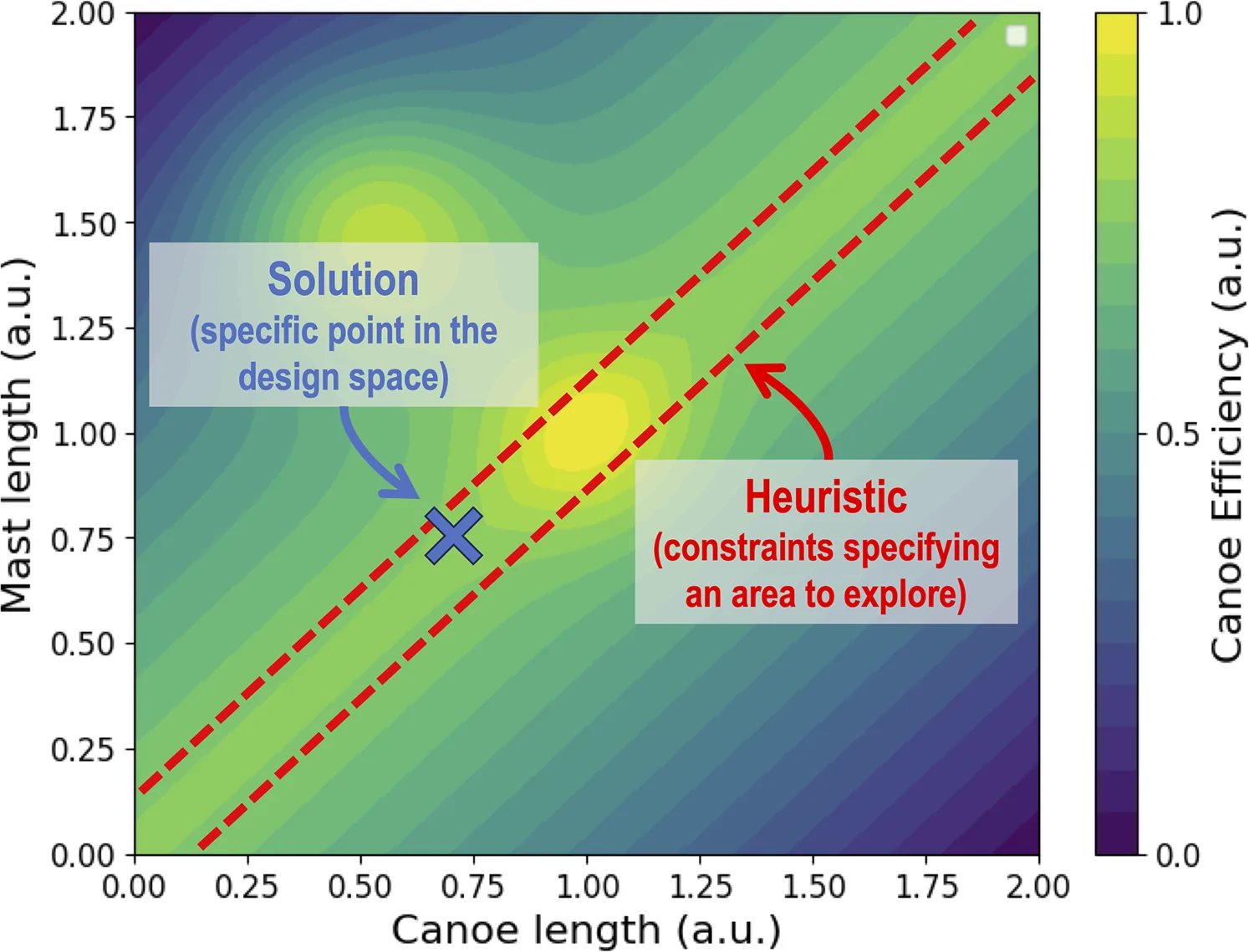
Hypothetical example of the effect of inheriting heuristics versus solutions receiving a solution provides the social learner with a better-than-average starting point in the cultural fitness landscape (e.g. a specific canoe design illustrated by the blue cross). Receiving a heuristic provides the social learner with an area of the fitness landscape that should be preferentially explored (e.g. specific proportions between mast length and canoe length, represented by the area between the two dotted red lines). Notice that inheriting a heuristic can lead to a smoother, more navigable fitness landscape. We expand on this observation in the discussion.

Beyond influencing exploration at the individual level, solutions and heuristics may also shape exploration at the group level by affecting the similarity between demonstrators’ and learners’ solutions. Consider, for instance, the rule ‘the length of the canoe should be equal to the length of the mast’. Receiving such a rule would probably lead social learners to produce solutions resembling their demonstrators’ in terms of canoe-to-mast length ratio, though not necessarily in the specific values of each dimension. We shall refer to this kind of similarity as structural similarity. By contrast, inheriting information about how to reproduce a specific design (e.g. a particular canoe design) would promote closer similarity with the demonstrator's solution in both canoe length and mast length (figure 1). We shall refer to this kind of similarity as visual similarity.

These differences between solutions and heuristics suggest that restricted exploration may not inherently result from receiving social information in general but may specifically arise when social information is provided in the form of a solution. In this paper, we test this hypothesis by examining how the cultural transmission of heuristics influences the generation of variation, both at the individual and intergenerational levels, compared with the transmission of solutions.

To address this question, we asked participants in transmission chains to solve a task representative of complex problem-solving scenarios, characterized by a large solution space that could be navigated more effectively by identifying regularities among parameters. The task involved assigning guests to rooms to maximize total guest satisfaction across 10 trials [18,19]. Guest satisfaction depended on two types of unknown preferences: room-based preferences (e.g. ‘guest A prefers room 1 over room 2’) and neighbour preferences (e.g. ‘guests A and B should not be in adjacent rooms’; figure 2a). Participants took part in three-generation transmission chains, with the first generation receiving no social information. Participants in subsequent generations received social information from the previous participant, either as a configuration (solution treatment) or as a heuristic constrained to the form ‘X and Y must be neighbours’ (heuristic treatment).

**Figure 2.**
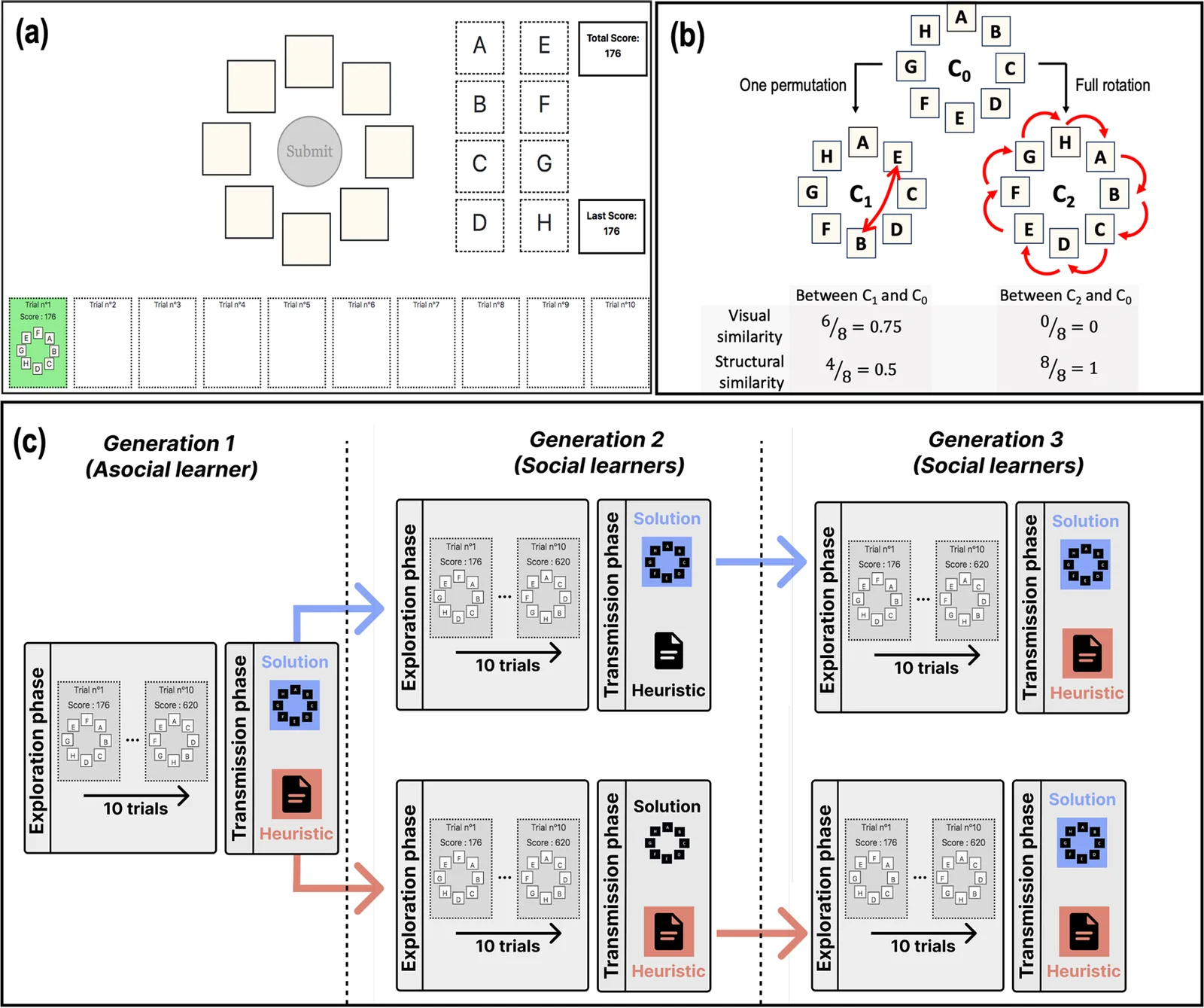
(a) The room assignment task. Screenshot of the task as presented to participants during the exploration phase. Guests (represented by the eight letters on the right side of the screen) had to be placed in rooms (represented by the eight empty squares in the middle of the screen). Participants could see their total score and their score at the last trial (right side of the screen), as well as the score of each previously submitted configuration (bottom of the screen). (b) Examples of visual and structural similarities. After a single permutation of guests B and E in configuration C_0_, the visual similarity with C_1_ is 6/8 (six guests are assigned to identical rooms in C_0_ and C_1_) and the structural similarity is 4/8 (four couples of guests are neighbours in both C_0_ and C_1_). After a full rotation of guests in C_0_, the visual similarity with C_2_ is 0/8 (none of the guests is assigned to the same rooms in C_0_ and C_2_) and the structural similarity is 8/8 (all pairs of neighbouring guests in C_2_ remain the same as in C_0_). (c) Structure of the transmission chains. Transmission chains had one participant in the first generation, two participants in the second generation and two participants in the third generation. Each participant produced two types of social information: a heuristic and a solution. First-generation participants did not receive any social information. Second-generation participants received either the solution or the heuristic—depending on their treatment—produced by the asocial learner. Third-generation participants received either the solution or the heuristic—depending on their treatment—produced by the second-generation participant from the same treatment.

To evaluate how the type of social information affects exploration of the design space, we defined two metrics capturing how similar two configurations are. We defined one measure of visual similarity and one measure of structural similarity (figure 2b). The visual similarity between two configurations is defined as the fraction of guests that are assigned to identical rooms in both configurations. The structural similarity between two configurations is defined as the fraction of pairs of guests that are neighbours in both configurations. While a high visual similarity indicates that two cultural products or behaviours are almost identical (e.g. two canoes that have a very similar design), a high structural similarity means that they share underlying structural features (e.g. two canoes that have the same length-to-mast ratio). These measures allowed us to quantify how broadly participants explored the design space, as well as how much they deviated from the previous generation. These metrics, as well as the experimental design, sampling procedure and analysis plan, were pre-registered before data collection [17]. Analyses that were not part of the pre-registration are explicitly labelled as exploratory.

## 2. Results

### (a) Inheriting heuristics leads to more frequent exploration

Participants who received a solution generate a lower number of unique configurations than those who received a heuristic (solution mean: 7.8, heuristic mean: 9.15, solution–heuristic contrast 89% credible intervals (CrI): [–1.87; –0.75]) and those who received no social information (asocial mean: 9.13, solution–asocial contrast 89% CrI: [–1.84; –0.75]; figure 3A). On the other hand, we did not detect any difference between participants who received a heuristic and those who did not receive any social information (heuristic–asocial contrast 89% CrI: [–0.53; 0.60]).

**Figure 3.**
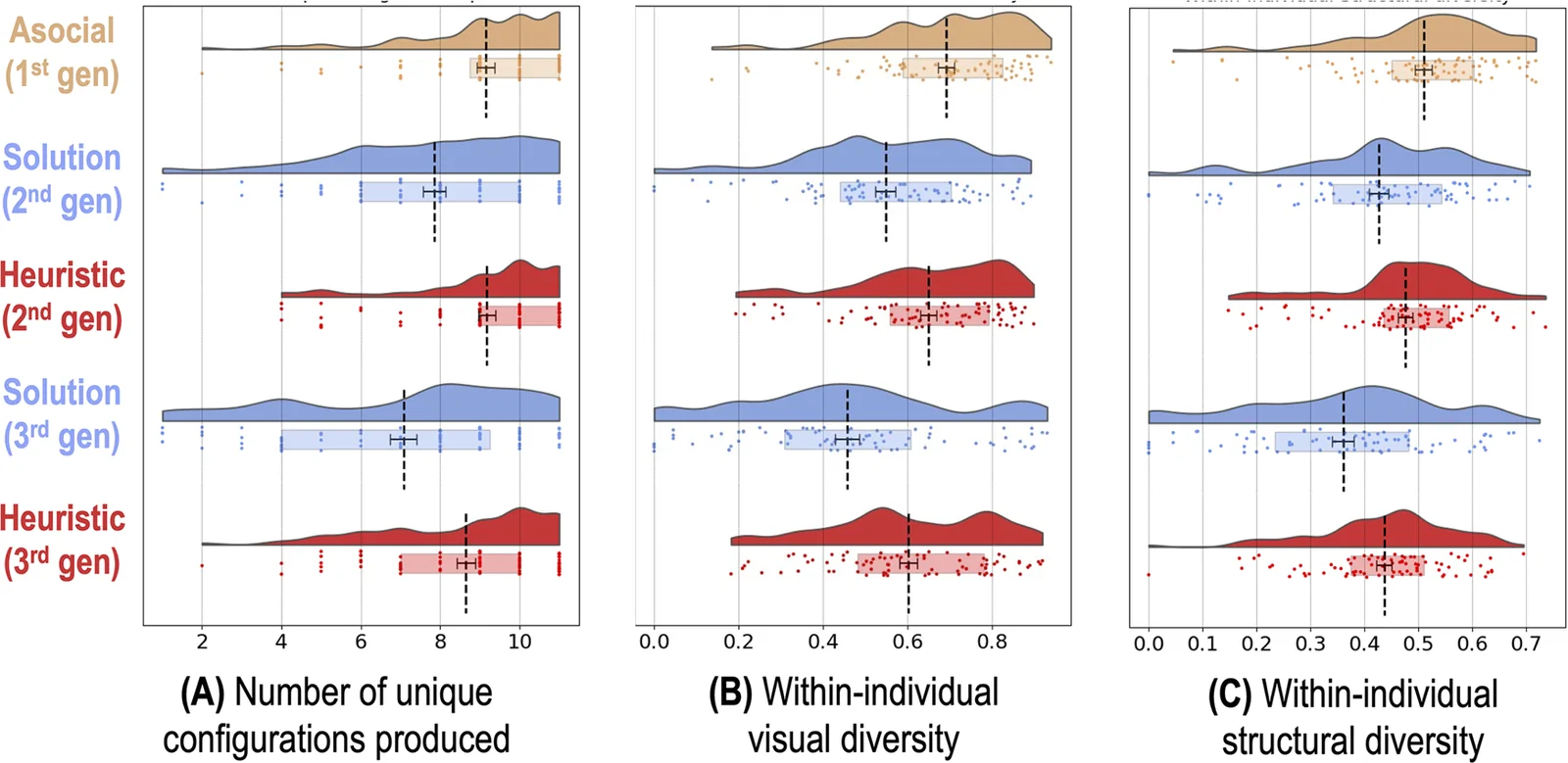
Participants who inherited a heuristic explored more often and less locally than participants who inherited a solution. Exploration measures for participants in the asocial (beige), solution (blue) and heuristic (red) treatments. Dashed black lines represent the mean for each treatment. The error bars represent the standard error of the mean. Rectangles represent the range from the first to the third quartile. (A) Number of unique configurations produced by each participant. Each participant is represented by a dot. (B) Within-individual visual diversity, defined as the average pairwise visual distance between solutions produced by a given participant. Each pair of solutions is represented by a dot. (C) Within-individual structural diversity, defined as the average pairwise structural distance between solutions produced by a given participant. Each pair of solutions is represented by a dot.

Exploratory analyses revealed that by the third generation, there was still no difference between the heuristic and the asocial treatment (heuristic-3rd generation mean: 8.64, heuristic-3rd generation–asocial contrast 89% CrI: [–1.06; 0.08]). By contrast, exploration continued to decrease for participants in the solution treatment (solution-3rd generation mean: 7.10, solution-3rd generation–solution-2nd generation contrast 89% CrI: [–1.32; –0.20]).

### (b) Inheriting heuristics leads to broader exploration

We quantified how social information influenced exploration by computing the average pairwise visual and structural similarity between configurations submitted by a participant, and averaged it over all pairs (figure 3B and C). Compared with participants in the asocial treatment, participants in both the solution and heuristic treatments explored more locally, displaying lower visual diversity (asocial mean: 0.69, solution mean: 0.55, heuristic mean: 0.65, solution–asocial contrast 89% CrI: [–0.17; –0.11]; heuristic–asocial contrast 89% CrI: [–0.07; –0.02]) and lower structural diversity (asocial mean: 0.51, solution mean: 0.43, heuristic mean: 0.48, solution–asocial contrast 89% CrI: [–0.10; –0.06]; heuristic–asocial contrast 89% CrI: [–0.06; –0.01]).

Participants in the heuristic treatment displayed higher diversity in their exploration than participants in the solution treatment, both in terms of visual diversity (heuristic–solution contrast 89% CrI: [0.07; 0.13]) and structural diversity (heuristic–solution contrast 89% CrI: [0.03; 0.07]).

Exploratory analyses revealed that this difference was maintained at the third generation, with participants in the heuristic treatment displaying broader exploration than participants in the solution treatment, both in terms of visual diversity (solution-3rd generation: 0.46, heuristic-3rd generation: 0.60, heuristic-3rd generation–solution-3rd generation contrast 89% CrI: [0.11; 0.17]) and structural diversity (solution-3rd generation mean: 0.37, heuristic-3rd generation: 0.44, heuristic-3rd generation–solution-3rd generation contrast 89% CrI: [0.05; 0.10]).

### (c) Inheriting heuristics leads to greater inter-generational variation

Receiving a solution led social learners to produce solutions more similar to that of their demonstrator compared with receiving a heuristic (figure 4), both in terms of visual similarity (solution mean: 0.58, heuristic mean: 0.15, heuristic–solution contrast 89% CrI :[–0.49; –0.37]) and structural similarity (solution mean: 0.67, heuristic mean: 0.39, heuristic–solution contrast 89% CrI: [–0.33; –0.23]). When receiving a heuristic, the visual similarity between social learners and asocial learners was at chance level (Chance 89% CrI: [0.12; 0.14] included in heuristic 89% CrI: [0.11; 0.19]), but structural similarity was above chance (chance 89% CrI: [0.26; 0.28] not overlapping with heuristic 89% CrI: [0.35; 0.42]).

**Figure 4.**
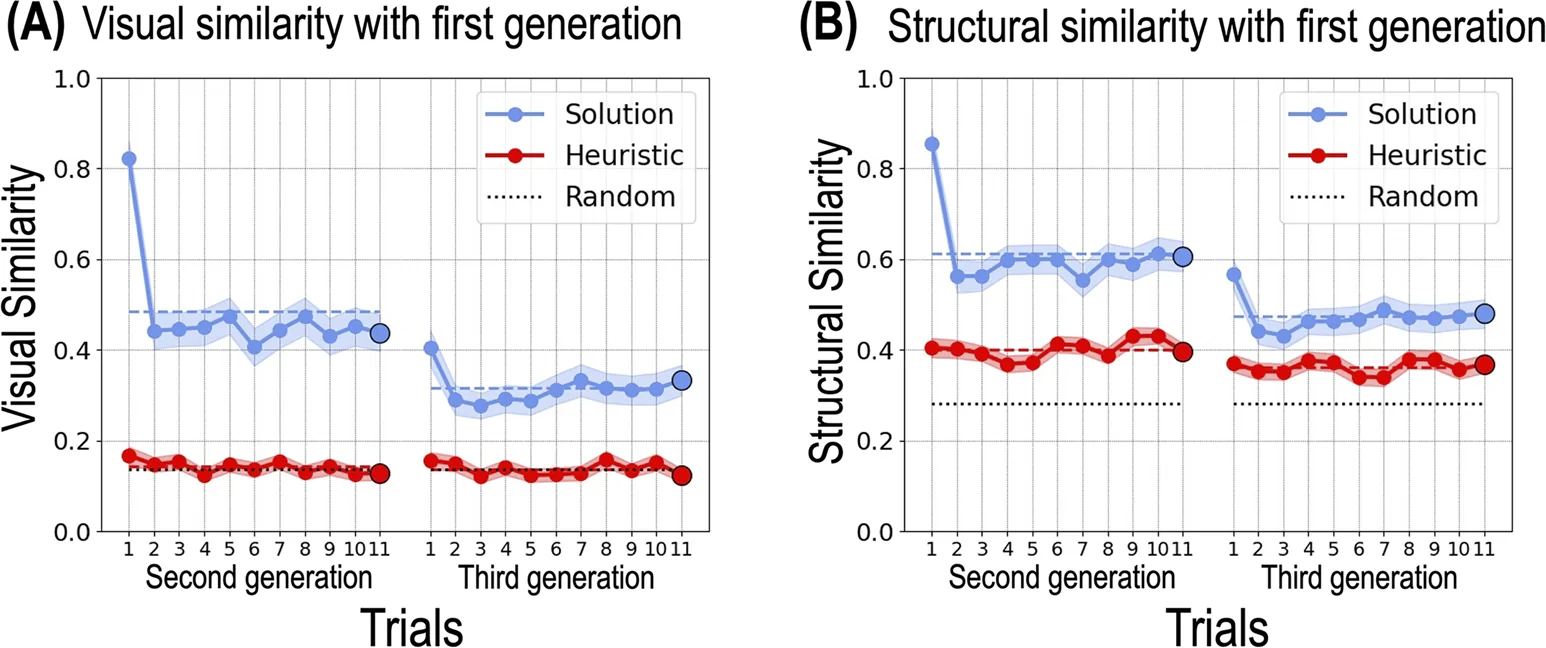
Social learners who inherited a heuristic, produce solutions that are less similar to those of their demonstrator. Inter-generational similarity for participants in the solution (blue) and heuristic (red) treatments, in both second and third generations. (A) Visual similarity between a configuration and the configuration transmitted by the asocial learner. (B) Structural similarity between a configuration and the configuration transmitted by the asocial learner. Coloured dots represent the mean over all participants of a given treatment at the corresponding trial. Dots corresponding to the configuration submitted during the transmission phase (trial 11) are represented by a larger dot with a black outline. Dashed coloured lines represent the mean for the corresponding treatment over all participants and all trials. The black dashed line represents the expected similarity between random configurations. The filled areas represent the standard error of the mean.

We also found evidence of an interaction between the effect of treatment and that of trial: both visual and structural similarities with the demonstrator decreased faster for participants in the solution treatment than for participants in the heuristic treatment (effect of trial on visual similarity: heuristic–solution contrast 89% CrI: [0.01; 0.02]; effect of trial on structural similarity: heuristic–solution contrast 89% CrI: [0.01; 0.02]). As can be seen in figure 4, this effect appears to be mainly driven by trial 1, after which similarity remains stable. This pattern likely stems from participants’ tendency to initially replicate the received solution before exploring alternative configurations.

Exploratory analyses revealed that for participants in the solution treatment, the similarity with the original demonstrator kept on decreasing at the third generation, with respect to both visual similarity (solution-3rd generation mean: 0.31, solution-3rd generation–solution-2nd generation contrast 89% CrI: [–0.22; –0.12]) and structural similarity (solution-3rd generation mean: 0.47, solution-3rd generation–solution-2nd generation contrast 89% CrI: [–0.18; –0.10]). For participants in the heuristic treatment, the average similarity to the original demonstrator did not differ between the second and third generations, neither in terms of visual similarity (heuristic-3rd generation mean: 0.14, heuristic-3rd generation–heuristic-2nd generation contrast 89% CrI: [–0.05; 0.05]) nor structural similarity (heuristic-3rd generation mean: 0.36, heuristic-3rd generation–heuristic-2nd generation contrast 89% CrI: [–0.08; 0.003]).

### (d) Both types of social information allow cumulative improvement

Whether they inherited a configuration or a heuristic, second-generation participants outperformed first-generation participants (figure 5A), with respect to both the total score obtained over all trials (asocial mean: 5156, solution mean: 6081, heuristic mean: 5959, solution–asocial contrast 89% CrI: [830.2, 1018.8]; heuristic–asocial contrast 89% CrI: [705.5, 894.7]) and the score obtained at the last trial (asocial mean: 502, solution mean: 547, heuristic mean: 527, solution–asocial contrast 89% CrI: [23.7, 65.4]; heuristic–asocial contrast 89% CrI: [5.3, 46.2]). Participants who received a solution obtained a slightly higher total score than participants who received a heuristic (heuristic–solution contrast 89% CrI: [–218.1; –31.3]), but we found no reliable difference when looking at the score obtained at the last trial (heuristic–solution contrast 89% CrI: [–40.1; 1.9]). This is consistent with participants from the solution treatment being more exploitative; they do not produce better solutions than those in the heuristic treatment, but they exploit their solutions more extensively.

**Figure 5.**
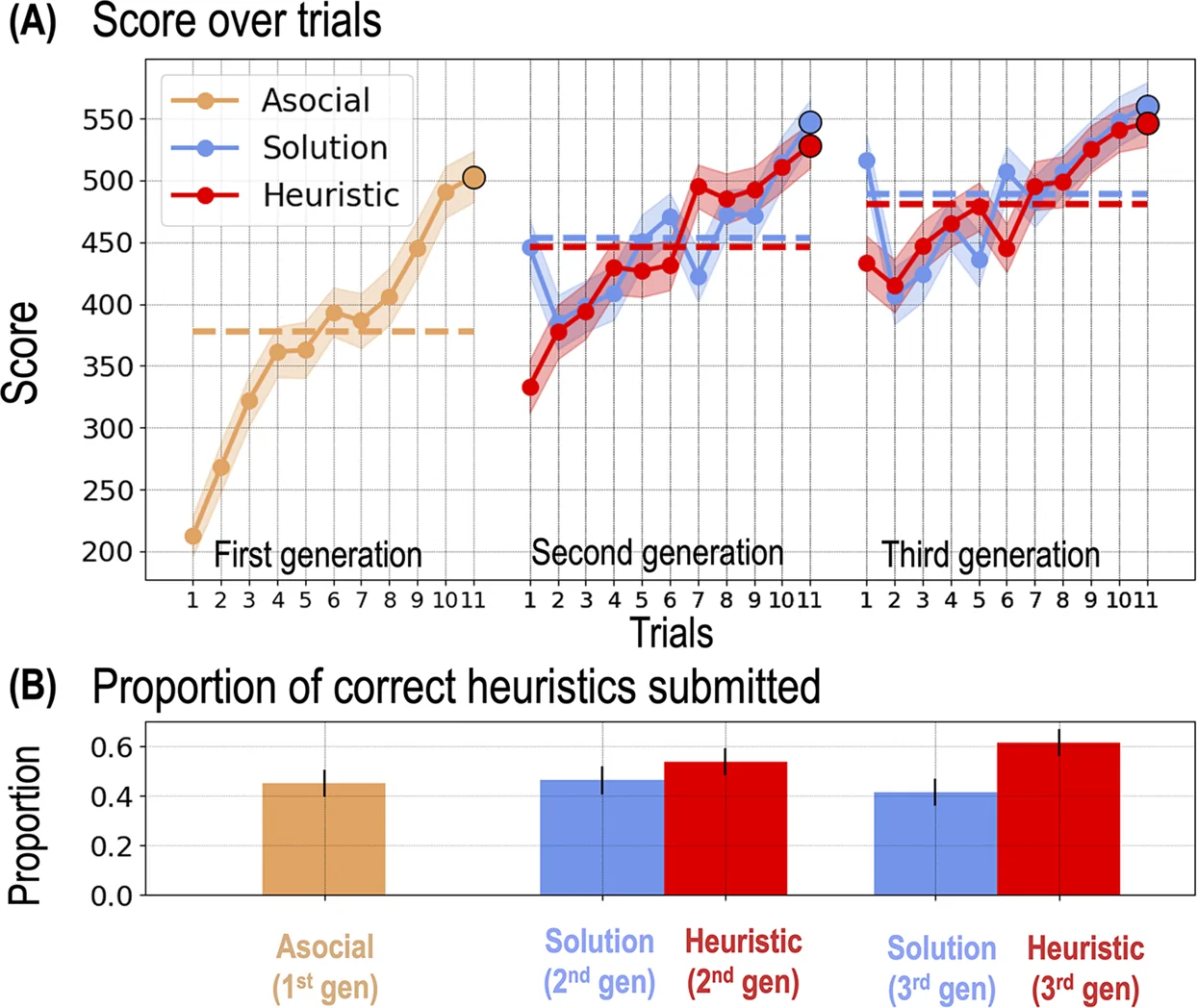
Receiving either a heuristic or a solution allows social learners to outperform their demonstrator. (A) Score at each trial of participants in asocial (beige), solution (blue) and heuristic (red) treatment. Coloured dots represent the mean of all participants in each treatment at the corresponding trial. Dots corresponding to the configuration submitted during the transmission phase (trial 11) are represented by a larger dot with a black outline. Dashed coloured lines represent the mean for the corresponding each treatment over all participants and all trials. The filled areas represent the standard error of the mean. (B) Proportion of participants who submitted a correct heuristic (i.e. a heuristic that increases the score when followed) during the transmission phase, for each treatment and generation. The height of the bars represents the proportion, and the error bars represent the standard error of the mean.

In exploratory analyses, we observed the same results at the third generation: participants who received a solution were slightly better with respect to their total score (solution-3rd generation mean: 6494, heuristic-3rd generation mean: 6376, heuristic-3rd generation–solution-3rd generation contrast 89% CrI: [–227.3; –6.6]), but there was no difference for the score at the last trial (solution-3rd generation mean: 5585, heuristic-3rd generation mean: 5449, heuristic-3rd generation–solution-3rd generation contrast 89% CrI: [–37.2; 9.8]).

In both treatments, participants’ total score kept on increasing at the third generation (solution-3rd generation–solution-2nd generation contrast 89% CrI: [308.48; 526.231]; heuristic-3rd generation–heuristic-2nd generation contrast 89% CrI: [302.843; 526.831]), but not their score at the last trial (solution-3rd generation–solution-2nd generation contrast 89% CrI: [–12.6; 35.0]; heuristic-3rd generation–heuristic-2nd generation contrast 89% CrI: [–4.9; 40.5]). None of the participants discovered the optimal configuration.

Additional exploratory analyses revealed that while both heuristics and solutions can support cumulative improvement, they might do so through different mechanisms. Indeed, the proportion of correct heuristics submitted by participants (figure 5B) increases over generations for participants in the heuristic treatment (heuristic-3rd generation–asocial contrast 89% CrI: [0.04, 0.27]), while it does not for participants in the solution treatment (solution-3rd generation–asocial contrast 89% CrI: [–0.16, 0.09]). This suggests that improvements might happen mainly through blind variation and retention when solutions are transmitted, while they may arise from an increasingly accurate representation of the problem when heuristics are transmitted.

## 3. Discussion

Social information is typically viewed as limiting exploration, yet prior research has primarily focused on the transmission of fully fledged solutions, overlooking other prevalent forms of information transfer. Here, we have addressed this gap by examining the relative effects of transmitting a solution versus a heuristic on the cultural variation generated by social learners. Our findings indicate that social learners who receive a heuristic produce greater cultural variation than those receiving a solution, demonstrating that the reduced exploration identified in previous works [7,9–11] does not extend to all forms of social information.

These results highlight a largely overlooked aspect of cultural transmission: different transmission mechanisms vary in their impact on learners’ exploration patterns. Culturally evolved solutions often provide naive learners with a better-than-average starting point in the parameter space. However, they offer little guidance for further exploration. Yet, during the process of developing those solutions, demonstrators may have uncovered useful regularities and structural properties of the search space. Transmitting only the final solution discards this acquired knowledge [20]. Heuristics, by contrast, can preserve and transmit such insights, serving as guides that support exploration and facilitate continued cultural innovation. For instance, the heuristic ‘avoid white mushrooms altogether’ [12] creates a new, more navigable search space by effectively removing particularly detrimental areas from the set of potential options. This view of heuristics as information about the search space is related to recent works arguing for the importance of culturally transmitted problem representations for open-ended cultural evolution [21–23]. These considerations also shed new light on the importance of verbal teaching for cultural evolution [24]. Indeed, teaching is generally thought to be central because it allows for faithfully replicating existing solutions—that is, it contributes to cultural stability [25,26]. Our results suggest that verbal teaching may also crucially contribute to cultural evolvability: while social learning mechanisms such as emulation or imitation only enable the transmission of the outcome of previous exploration, verbal teaching allows providing information about the properties of the search space—for instance, by transmitting heuristics.

The transmission of heuristics creates an interesting complementarity between social learning and individual learning. When solutions are transmitted, the two processes operate largely independently: having access to an efficient, previously discovered solution is beneficial regardless of the learner's subsequent exploration. By contrast, the transmission of heuristics is only effective when combined with the social learner's capacity for trial-and-error learning. This is clearly illustrated in our results: participants who inherited a heuristic began with lower performance but, through individual learning, gradually caught up to the performance of those who had received a solution. This view aligns closely with two recent models of cultural evolution. In the first [27], the authors address the challenge of transmitting tacit knowledge, demonstrating that expert knowledge can be used to reorganize the problem space so that trial-and-error learning reliably converges on a correct solution. In the second [28], the authors model the transmission of analogies and suggest that ‘teachers guide learners to the solution to a novel problem by invoking similarities to known problems and their known solutions’ [28]. In both models, as in our experiment, cultural transmission allows learners to engage in socially guided, efficient exploration, rather than enabling them to faithfully replicate their demonstrator's solution.

That faithful replication is not crucial to the efficiency of social learning is further supported by our results on inter-generational variation. While social learners who received a solution produced outputs closely resembling those of their demonstrator, participants who received a heuristic exhibited only limited structural similarity and no visual similarity with their demonstrator's solution. Despite this low inter-generational similarity, our analysis of the scores revealed that transmitting heuristics, just like transmitting solutions, was sufficient to observe cumulative improvement. Moreover, we found no difference between those two treatments with respect to the efficiency of the last solution produced by social learners. These findings suggest that high fidelity between teacher and learner outputs is not a necessary condition for cumulative improvement. Instead, they highlight the equally important role of cultural transmission in fostering socially guided, efficient exploration.

It appears important to make one clarification here. Although our results suggest that faithful replication of cultural *products* is not necessary for cumulative culture, this does not imply that high-fidelity transmission of cultural *information* is not important. Actually, our experimental design assumes perfect transmission of cultural information: solutions and heuristics are both transmitted to the social learner without errors. It is therefore crucial to make the distinction between cultural information and cultural products; a low similarity between two cultural products does not necessarily correspond to a large difference between the cultural information that led to their productions. In our case, two individuals employing the same heuristic (‘cultural information’) may produce very different configurations (‘cultural product’). The issue here arises from what has been described as the *relevance problem* [29], which describes the difficulty in determining which are the relevant dimensions along which to compute similarity. In our case, visual similarity between cultural products is irrelevant for inferring the transmission of heuristics, while it is relevant for identifying the transmission of solutions. The relevance problem, which is clearly illustrated in our study, might be especially important for researchers aiming to infer cultural dynamics from the archaeological record [30,31].

While our study focused on the capacity of heuristics to sustain exploration, it opens several avenues for future research. First, a natural extension of the current work would be to evaluate the advantage of heuristics relative to solutions in changing environments. Indeed, a key feature of heuristics is their flexibility [13,32]. When a social learner inherits a solution that becomes ineffective or obsolete owing to environmental change, they are left with little guidance for generating a new one. For example, a learner taught exactly where to forage for mushrooms may struggle to adjust if that specific patch becomes unavailable. By contrast, a heuristic such as ‘the older the forest, the more mushrooms’ [13] offers a transferable principle that can be applied flexibly to novel situations. Our results support the view that the transmission of heuristics may be more beneficial than the transmission of solutions in variable environments: social learners who received a solution tended to explore locally around the inherited configuration, which may hinder adaptation if a previously optimal solution becomes maladaptive. By contrast, the broader exploratory behaviour observed among participants who received a heuristic may offer a better foundation for responding to environmental change. This prediction is also supported by [28], which shows that the evolution of socially guided individual learning is favoured in variable environments.

Besides environmental change, heuristics may also be advantageous in situations where there exists inter-individual variation with respect to what constitutes an adequate cultural trait. For instance, the use of heuristics has been reported for guiding the construction of kayaks (‘the total length of the kayak should be approximately 3 times the height of the kayaker’ [33]), addressing the fact that the optimal dimensions of a kayak depend on the size of the individual who uses it. This links with a second fruitful avenue for future research, namely the cultural evolution of heuristics themselves. In our experimental design, participants were explicitly asked to provide a heuristic in a specific format. Those heuristics targeted structural properties of the search space, and were simple to understand and to apply, in line with most reported examples of heuristics. It would, however, be very informative to study how heuristics come to acquire such features, and how their evolution is jointly determined by selection for function—targeting relevant variables and selection for fidelity—being easy to remember and apply [34]. This line of research could also help clarify the conditions under which heuristics become the preferred pathway of cultural inheritance.

Overall, although most empirical studies have focused on the transmission of solutions, our study suggests that guiding the exploratory behaviour of social learners by transmitting heuristics may be sufficient to observe cumulative improvements of cultural products. We show that the transmission of such heuristics is likely to have very different consequences on exploratory behaviours and cultural variation compared with the transmission of solutions. This calls for more investigation of the different types of knowledge that can be culturally transmitted, and on how they differentially influence cultural evolutionary dynamics.

## 4. Materials and methods

### (a) Participants

A total of 418 adult participants took part in the study. Eighteen participants were excluded from the analysis according to pre-registered exclusion criteria, either because of technical issues (17 participants) or because they failed the attention check (one participant). Analyses were therefore performed on 400 participants, which was the pre-registered targeted sample size (mean age = 29.74; s.d. = 10.04; women: 195; men: 199; other: 6).

The experimental protocol was approved by the ethics committee of IAST. All participants provided informed consent by checking a box before beginning the study. Experimental design, sampling procedure and analysis plan were pre-registered before data collection [17].

### (b) Task description

Participants completed the task online using a web interface programmed using *oTree* [35]. Eight guests (represented by letters from A to H) had to be placed in eight rooms, with each room containing exactly one guest (figure 2a). Participants were informed that each guest has preferences regarding the room in which they sleep, and that some but not all guests have strong preferences regarding who their neighbours are. However, participants were not told what those preferences were. To submit a configuration, participants used a drag-and-drop interface to assign each guest to a room. After submitting a configuration, participants were informed about the score associated with this configuration, i.e. the summed preferences of guests (see supplementary material for details about score calculation).

The experiment contained two successive phases (figure 2c).

— Exploration phase: participants completed ten trials, submitting one configuration per trial. After each submission, they received feedback in the form of a score (figure 2a). They could see the history of all configurations they had already submitted and the score associated with each configuration. This phase ended once participants had submitted 10 configurations. Participants were incentivized to maximize their cumulative score over those 10 trials.
— Transmission phase: participants were asked to provide a heuristic specifying a detected regularity, constrained to the form ‘X and Y must be neighbours’, where they could only choose the values of X and Y. They were informed that providing an accurate heuristic (i.e. a heuristic that indeed has a positive impact on guest satisfaction) would increase their bonus payment. During this phase, they could see the 10 configurations they submitted during the previous phase and their associated scores. After they had submitted a heuristic, they were asked to submit an additional configuration. Participants were informed that the score associated with this configuration would be multiplied by three to incentivize them to submit what they believed to be the best configuration. The *heuristic* and *solution* produced during this phase constitute the two types of social information that would then be transmitted to the next participants.

### (c) Treatments

We used a modified version of the transmission chain paradigm [36–38]. Participants were assigned to one of three treatments (*asocial*, *solution* and *heuristic*) and were attributed a position in a chain. Chains were three generations long, but involved five participants: one participant in the first position, two participants in the second position and two participants in the third position (figure 2c).

Participants in the first position were all part of the *asocial* treatment. They did not receive any social information and completed the task exactly as described above.

For each chain, one of the participants in the second position was assigned to the *solution* treatment, and the other participant in the second position was assigned to the *heuristic* treatment. In the same way, one of the participants in the third position was assigned to the *solution* treatment, and the other participant in the third position was assigned to the *heuristic* treatment. Second-generation participants received either the solution or the heuristic—depending on their treatment—produced by the asocial learner. Third-generation participants received either the solution or the heuristic—depending on their treatment—produced by the second-generation participant from the same treatment (figure 2b).

Participants in both the *solution* and *heuristic* treatments were informed that a participant had taken part in the exact same experiment before them. Participants in the *solution* treatment were told that they would receive the last configuration submitted by the previous participant. Participants in the *heuristic* treatment were told that they would receive the heuristic submitted by the previous participant. In both treatments, social information remained visible throughout the whole exploration phase.

### (d) Analyses

#### (i) Visual and structural similarity measures

We defined two metrics capturing how similar two configurations are. We defined one measure of *visual similarity* and one measure of *structural similarity*. The *visual similarity* between two configurations is defined as the fraction of guests that are assigned to identical rooms in both configurations. The *structural similarity* between two configurations is defined as the fraction of pairs of guests that are neighbours in both configurations. Figure 2b provides examples of visual and structural similarities between different configurations.

#### (ii) Individual exploration measures

To quantify frequency of exploration at the individual level, we counted the number of unique configurations submitted by participants among the 11 configurations submitted in total. To quantify breadth of exploration at the individual level, we computed the pairwise visual and structural similarities between configurations submitted by a participant, and averaged it over all pairs. We use this value to define within-individual visual (or structural) diversity as 1−(average pairwise visual (or structural) similarity).

#### (iii) Statistical analyses

Data analysis was performed using R v. 4.3.1 [39]. We used Bayesian multi-level models to conduct statistical analyses. Models were fitted using the *Statistical Rethinking* package [40]. We used samples from the posterior to compute contrasts between treatments. Eighty-nine percent credible intervals of the posterior contrasts are reported. Evidence for an effect was inferred when the 89% credible interval excluded 0. The choice of 89% credible intervals, which was pre-registered, is intended to avoid triggering a frequentist interpretation (where 95% confidence intervals are routinely used) and to highlight that the cut-off is arbitrary (95% is not more justified than 89%, see [40]). Unless explicitly labelled as exploratory, all statistical models were registered on OSF prior to data collection [17]. Full details of the statistical models are provided in the supplementary material.

## Supplementary Material



## Data Availability

Data and code are publicly available at Zenodo [41]. Supplementary material is available online [42].
