# Supplementary material for: Socially guided individual learning mitigates the double-edged sword of pedagogy
Source: Proc Biol Sci. 2026 Oct 14;293(2080):20260619. doi: 10.1098/rspb.2026.0619 (PMC13678623; doi:10.1098/rspb.2026.0619)
Supplement: Supplementary file 1 [file rspb.2026.0619.zip › rspb.2026.0619/rspb20260619_si_001.pdf]

(1/2) Supplementary Materials for

Socially Guided Individual Learning Mitigates the Double-Edged Sword of Pedagogy

DOI: 10.1098/rspb.2026.0619

Authors:
Jérémy Perez[1,2], Maxime Derex[2]

Affiliations:
[1]Flowers AI & CogSci Lab, Centre INRIA de l'Université de Bordeaux, Talence, France
[2]Institute for Advanced Studies in Toulouse, Toulouse, France

1) Additional details on the methods

Participants recruitment
Participants were recruited by posting the online task on Prolific, which is an online platform that centralizes the recruitment of participants for online experiments and surveys. From Prolific, participants accessed a URL that directed them to the web-page of the study. The only requirements for selecting participants were that they were at least 18 years old and fluently speak English. Participants received £1.6 for completing the task and a bonus payment of up to £1.25 (mean = £0.77, SD = £0.27) depending on their performance (see bonus payment section below). Participants' age ranged from 18 to 68 (mean = 29.74; SD = 10.04). The sex indicated by users on Prolific was used to balance participants' sex between treatment.

Pre-experiment information and tutorial
Before starting the experiment, participants were provided with information about the task (Figures S1 and S3). They were told that they would have to solve a room assignment problem, which would involve assigning 8 guests to 8 individual rooms. They were informed that the satisfaction of all guests depended on the room in which they slept, and that for some guests, who their neighbors are would tly impact their satisfaction. They were told that they would have to maximize the satisfaction of the guests over N trials, and that their bonus payment would depend on the cumulated score. They were also informed that they had no time limit to complete the task. Participants were then asked to submit a test configuration to verify that they were able to use the drag-and-drop interface. They also had to answer a comprehension test of four questions (Figure S2) to verify that they understood and memorized the information provided.

Score Calculation
The score associated with a given configuration depended on the total satisfaction of the guests. The satisfaction of all guests depended on the room to which they were assigned. For each room-guest pair, we draw uniformly at random a value between 0 and 50 to determine the satisfaction of this guest when assigned to this room. These values were randomly generated before the experiment, but were then fixed and identical for all participants. Additionally, when guests A and C were placed in neighboring rooms, the total satisfaction

associated with the configuration was increased by 240. Similarly, when guests B and D were placed in neighboring rooms, the total satisfaction was increased by 240. The pairs A-C and B-D were chosen following pilot studies which revealed that they ensured that some participants, but not all, would discover them during the Exploration phase. The best configuration yielded a score of 931.

Bonus Payment

To compute the amount of the payment awarded to participants, we divided the total score obtained over all trials (including the bonus trial which counted triple) by 9310 (corresponding to 10 times the maximal possible score on one configuration). This resulted in bonus payments ranging from £0.22 to £1.25 (mean: 0.77; std: 0.26). Bonus payment were then increased by £0.25 if the participant submitted an accurate heuristic. This total value represented the amount of their bonus payment in pounds.

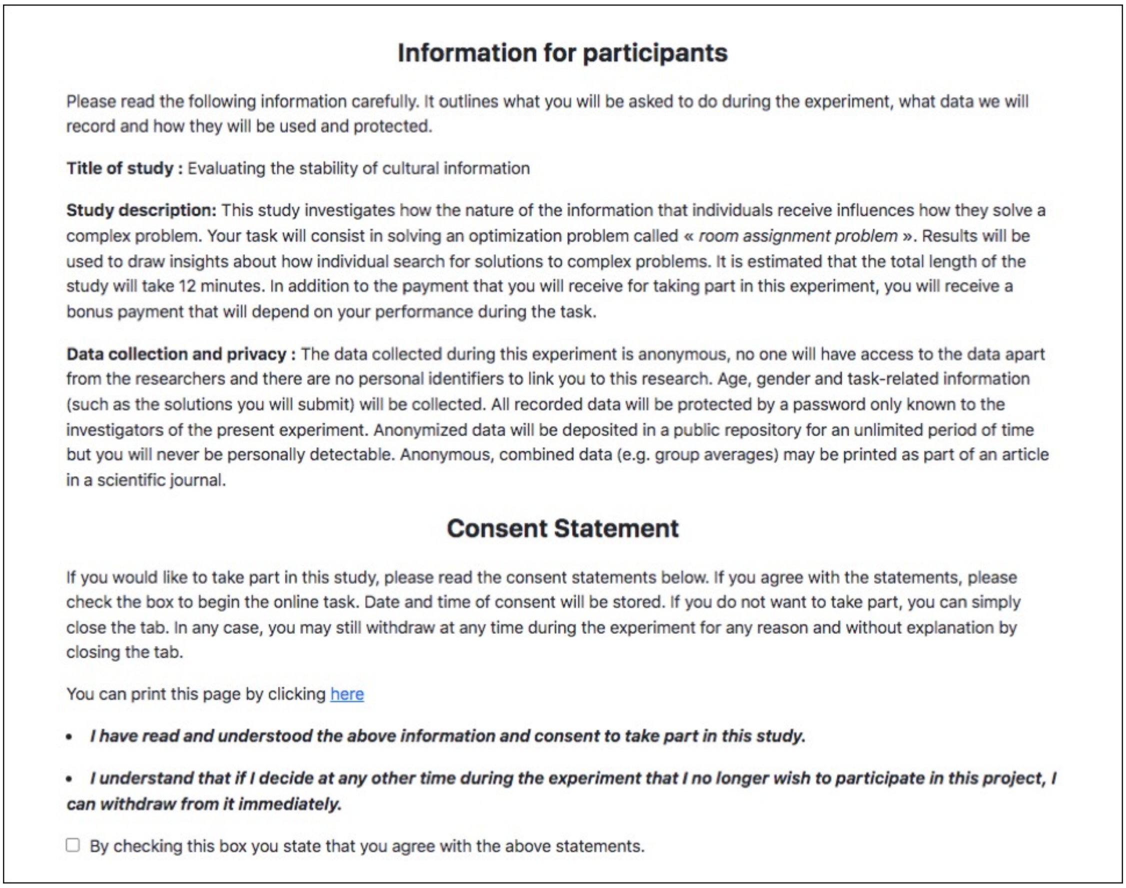

Information for participants

Please read the following information carefully. It outlines what you will be asked to do during the experiment, what data we will record and how they will be used and protected.

Title of study : Evaluating the stability of cultural information

Study description: This study investigates how the nature of the information that individuals receive influences how they solve a complex problem. Your task will consist in solving an optimization problem called « *room assignment problem* ». Results will be used to draw insights about how individual search for solutions to complex problems. It is estimated that the total length of the study will take 12 minutes. In addition to the payment that you will receive for taking part in this experiment, you will receive a bonus payment that will depend on your performance during the task.

Data collection and privacy : The data collected during this experiment is anonymous, no one will have access to the data apart from the researchers and there are no personal identifiers to link you to this research. Age, gender and task-related information (such as the solutions you will submit) will be collected. All recorded data will be protected by a password only known to the investigators of the present experiment. Anonymized data will be deposited in a public repository for an unlimited period of time but you will never be personally detectable. Anonymous, combined data (e.g. group averages) may be printed as part of an article in a scientific journal.

Consent Statement

If you would like to take part in this study, please read the consent statements below. If you agree with the statements, please check the box to begin the online task. Date and time of consent will be stored. If you do not want to take part, you can simply close the tab. In any case, you may still withdraw at any time during the experiment for any reason and without explanation by closing the tab.

You can print this page by clicking here

- ***I have read and understood the above information and consent to take part in this study.***
- ***I understand that if I decide at any other time during the experiment that I no longer wish to participate in this project, I can withdraw from it immediately.***

☐ By checking this box you state that you agree with the above statements.

Fig. S1. Pre-experiment information and consent form

Comprehension Test

Please answer the following questions to make sure that you understood the instructions.

1) What can influence the satisfaction of the guests ?

- ○ Who their neighbors are
- ○ In which room they sleep
- ○ Both answers

2) What determines the amount of your bonus payment ?

- ○ The maximal score I achieve in a single night
- ○ The total score I accumulate over the 10 nights

3) Select the correct answer :

- ○ All guests have preferences regarding the room in which they sleep
- ○ A few guests have preferences regarding the room in which they sleep
- ○ None of the guests have preferences regarding the room in which they sleep

4) Select the correct answer :

- ○ All guests have preferences regarding who their neighbours are
- ○ A few guests have preferences regarding who their neighbours are
- ○ None of the guests have preferences regarding who their neighbours are

Next Page

Fig. S2. Comprehension test

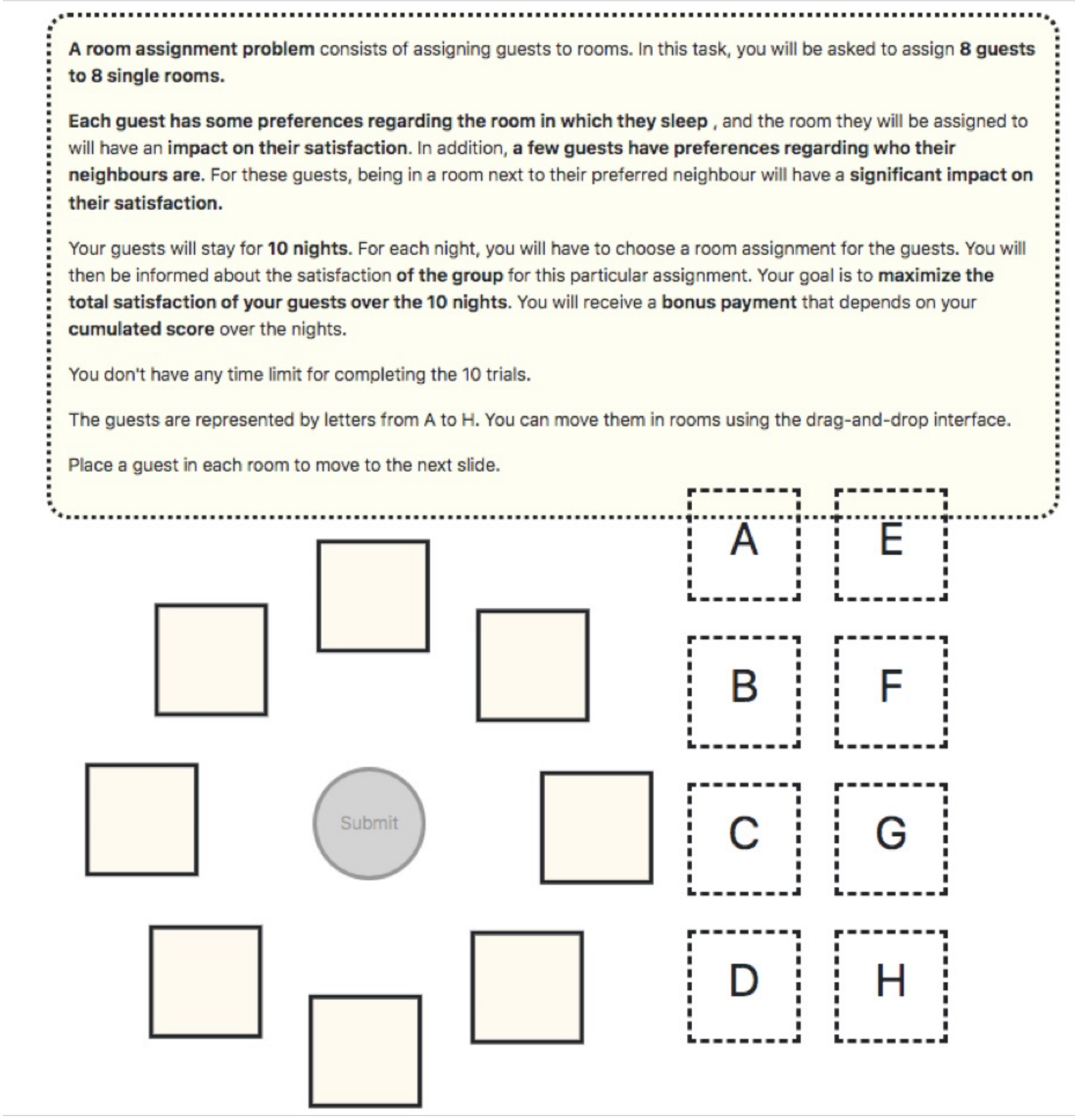

Fig. S3. Pre-experimental tutorial

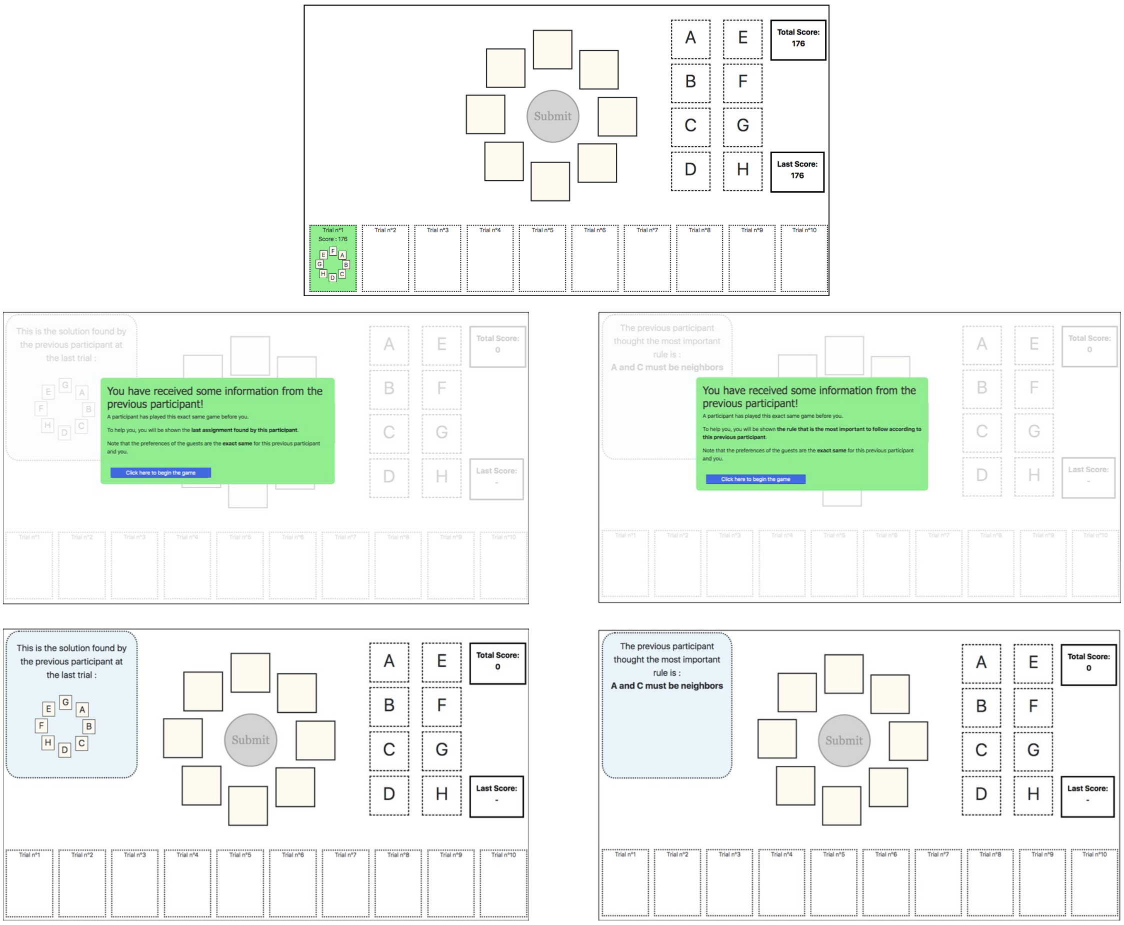

Fig. S4. Screenshots of the Exploration Phase

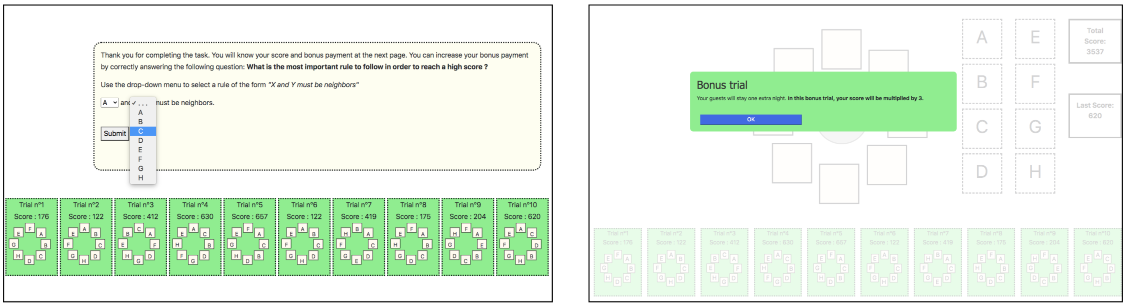

Fig. S5. Screenshots of the Transmission Phase
