# Supplementary material for: Socially guided individual learning mitigates the double-edged sword of pedagogy
Source: Proc Biol Sci. 2026 Oct 14;293(2080):20260619. doi: 10.1098/rspb.2026.0619 (PMC13678623; doi:10.1098/rspb.2026.0619)
Supplement: Supplementary file 1 [file rspb.2026.0619.zip › rspb.2026.0619/rspb20260619_si_002.pdf]

(2/2) Supplementary Materials for

Socially Guided Individual Learning Mitigates the Double-Edged Sword of Pedagogy

DOI: 10.1098/rspb.2026.0619

Authors:
Jérémy Perez[1,2], Maxime Derex[2]

Affiliations:
[1]Flowers AI & CogSci Lab, Centre INRIA de l'Université de Bordeaux, Talence, France
[2]Institute for Advanced Studies in Toulouse, Toulouse, France

2) Statistical models

Statistical models for pre-registered analyses were published on OSF (*https://osf.io/gxvnq*) prior to data collection.

Pre-registered model 1: Effect of social information on exploration frequency
To investigate the effects of the two types of social information on exploration, we studied the number of different configurations submitted by each participant. This value ranges between 1 (all configurations submitted by the participant are identical) to 11 (all configurations submitted by the participant are different). We fitted a linear model predicting Exploration (number of different configurations) as a function of treatment (0: Asocial, 1: Configuration second-generation; 2: Rule second-generation).

Equation:
Number of unique configurations $\sim \mathrm{N}(\mu_i, \sigma)$

$$\mu_i = a_{\text{treatment}[i]}$$

Priors:

$$a_{\text{treatment}} \sim \text{Normal}(8,2)$$
$$\sigma \sim \text{Exponential}(1)$$

Pre-registered model 2: Effect of social information on exploration breadth
To investigate the effects of the two types of social information on the variation generated by participants, we studied the visual and structural distances between configurations submitted by each participant. We computed the visual and structural distance between each pair of solutions, and summed these distances to obtain a measure of variation for each participant. We fitted a linear model predicting Aggregated visual Variation (summed visual distances between all pairs of configurations) as a function of treatment (0: Asocial, 1: Configuration second-generation; 2: Rule second-generation). Similarly, we fitted a linear model predicting Aggregated Structural variation (summed structural distances between all pairs of configurations) as a function of treatment.
Note: We use the term "diversity" instead of "variation" in the main text for better interpretability.

Equation:

visual variation $\sim \mathrm{N}(\mu_i, \sigma)$

$$\mu_i = a_{\text{treatment}[i]}$$

Priors:

$$a_{\text{treatment}} \sim \text{Normal}(250{,}30)$$
$$\sigma \sim \text{Exponential}(10)$$

Equation:

Structural variation $\sim \mathrm{N}(\mu_i, \sigma)$

$$\mu_i = a_{\text{treatment}[i]}$$

Priors: $a_{\text{treatment}} \sim \text{Normal}(250{,}30)$

$$\sigma \sim \text{Exponential}(10)$$

Pre-registered model 3: Effect of social information on inter-generation variation

To investigate the effect of the type of social information on inter-generational variation, we studied the distance between a solution produce by a second-generation participant at a given trial with the last solution produced by the first-generation participant of the same chain. To do so, we computed the visual and structural distances between each configuration produced by a participant with the last configuration submitted by the previous participant. We fitted two linear models (one for the visual distance, and one for the structural distance) predicting distance as a function of treatment (1: Configuration second-generation; 2: Rule second-generation) and trial number (0-11), controlling for participant identity. To determine whether the first configuration produced by second-generation participants resembles the last solution produced by first-generation participants, we estimated the distance (both visual and structural) between randomly generated configurations. If the confidence interval of the distance between random configurations falls completely inside the confidence interval of the effect of a treatment, this treatment is considered as having no effect on the distance. If this confidence interval for random configurations falls neither completely inside nor completely outside the estimated confidence interval for a treatment, we increased the number of random configurations generated until it falls either completely inside or completely outside.
Note: We report "similarity" instead of "distance" in the main text for better interpretability. This is a deterministic, monotonic linear transformation (similarity = 1 – distance / 8), which thus preserves the shape of the posterior and leads to identical statistical conclusions.

Equation:

Visual distance $\sim \mathrm{N}(\mu_i, \sigma)$

$$\mu_i = a_{\text{treatment}[i]} + b_{\text{trial}[\text{treatment}[i]]} \cdot \text{trial}_i + d_{\text{p\_ID}} \cdot \sigma_{\text{participant}}$$

Priors:

$$a_{\text{treatment}} \sim \text{Normal}(250{,}30)$$
$$b_{\text{trial}[\text{treatment}]} \sim \text{Normal}(0{,}30)$$
$$d_{\text{pID}} \sim \text{Normal}(0{,}1)$$
$$\sigma_{\text{participant}} \sim \text{Exponential}(1)$$
$$\sigma \sim \text{Exponential}(10)$$

Equation:

Structural distance $\sim \mathrm{N}(\mu_i, \sigma)$

$$\mu_i = a_{\text{treatment}[i]} + b_{\text{trial}[\text{treatment}[i]]} \cdot \text{trial}_i + d_{\text{p\_ID}} \cdot \sigma_{\text{participant}}$$

Priors:

$$a_{\text{treatment}} \sim \text{Normal}(250,30)$$
$$b_{\text{trial[treatment]}} \sim \text{Normal}(0,30)$$
$$d_{\text{pID}} \sim \text{Normal}(0,1)$$
$$\sigma_{\text{participant}} \sim \text{Exponential}(1)$$
$$\sigma \sim \text{Exponential}(10)$$

Pre-registered model 4: Effect of social information on task performance

To investigate whether transmitting a heuristic can support cumulative improvement of solutions, we evaluated the effect of the treatment on the total score obtained over all trials, and on the score obtained at the bonus trial. Treatment was coded as a categorical variable where 1 corresponds to the Asocial treatment, 2 to the Configuration treatment, and 3 to the Rule treatment. Data for the Configuration and Rule treatment was restricted to participants in second position of the chain (as pre-registered). We fitted a linear model predicting Total Score as a function of the treatment, and another linear model predicting Last Score as a function of the treatment.

Equation:

$Total\ score \sim \text{N}(\mu_i, \sigma)$

$\mu_i = a_{\text{treatment}[i]}$

Priors:

$$a_{\text{treatment}} \sim \text{Normal}(4000,1000)$$
$$\sigma \sim \text{Exponential}(10)$$

Equation:

$Score \sim \text{N}(\mu_i, \sigma)$

$$\mu_i = a_{\text{treatment}[i]}$$

Priors:

$$a_{\text{treatment}} \sim \text{Normal}(400,100)$$
$$\sigma \sim \text{Exponential}(10)$$

Exploratory analyses including third generation

In exploratory analyses, we included the third generation of participants in the analyses. The *Treatment* variable had thus 5 possible values: 1 corresponds to the *Asocial* treatment, 2 to the *Solution-2ndGeneration* treatment, 3 to the *Heuristic-2ndGeneration*, 4 to the *Solution-3rdGeneration* treatment and 5 to the *Heuristic-3rdGeneration*. We then ran the 7 pre-registered models described above with these 5 categories for *Treatment.*

Exploratory analyses about the proportion of correct heuristics

We conducted exploratory analyses to investigate whether the accuracy of the heuristics submitted by participants differed between the Solution and the Heuristic treatment. The accuracy of a heuristic is coded as 1 if it indeed has a positive impact on guest satisfaction, and 0 otherwise. There are 4 heuristics which have positive impacts on guest satisfactions: "A and C should be neighbours", "C and A should be neighbours", "B and D should be neighbours" and "D and B should be neighbours".

We fitted a linear model predicting heuristic accuracy as a function of the treatment (Solution / Heuristic).

Equation:

$$Accuracy \sim \mathrm{Binom}(1,\mathrm{p})$$
$$logit(p) \quad = a_{\mathrm{treatment}}$$

Priors:

$$a_{\mathrm{treatment}} \sim \mathrm{Normal}(0,1.5)$$

Output of statistical models

We report for each model the 89% credible intervals for each estimated parameter, as well as for contrasts between parameters, in supplementary Figures S6, S7 and S8.

	mean	sd	5.5%	94.5%
a[Asocial]	9.133	0.245	8.749	9.522
a[Solution]	7.845	0.235	7.472	8.22
a[Heuristic]	9.153	0.256	8.739	9.575
sigma	2.147	0.098	1.998	2.313
a[Solution] – a[Asocial]	–1.288	0.337	–1.844	–0.747
a[Heuristic] – a[Asocial]	0.02	0.358	–0.531	0.595
a[Heuristic] – a[Solution]	1.308	0.35	0.746	1.868

Model 1: Number of unique configurations submitted

	mean	sd	5.5%	94.5%
a[Asocial]	0.687	0.012	0.668	0.706
a[Solution]	0.547	0.012	0.528	0.566
a[Heuristic]	0.646	0.012	0.626	0.665
sigma	47.09	1.031	45.474	48.66
a[Solution] – a[Asocial]	–0.14	0.017	–0.166	–0.112
a[Heuristic] – a[Asocial]	–0.041	0.016	–0.067	–0.015
a[Heuristic] – a[Solution]	0.099	0.017	0.072	0.126

Model 2: Within-Individual visual diversity

	mean	sd	5.5%	94.5%
a[Asocial]	0.512	0.01	0.496	0.527
a[Solution]	0.43	0.01	0.413	0.446
a[Heuristic]	0.478	0.01	0.463	0.493
sigma	38.46	0.972	36.905	40.032
a[Solution] – a[Asocial]	–0.082	0.014	–0.104	–0.06
a[Heuristic] – a[Asocial]	–0.034	0.014	–0.056	–0.012
a[Heuristic] – a[Solution]	0.048	0.014	0.025	0.071

Model 3: Within-Individual structural diversity

	mean	sd	5.5%	94.5%
a[Solution]	0.582	0.027	0.537	0.625
a[Heuristic]	0.153	0.025	0.112	0.192
b[Solution]	0.983	0.002	0.979	0.986
b[Heuristic]	0.998	0.002	0.994	1.001
sigma	1.73	0.031	1.681	1.779
a[Chance]	0.13	0.006	0.12	0.14
a[Heuristic] – a[Solution]	–0.429	0.037	–0.489	–0.37
b[Heuristic] – b[Solution]	0.015	0.003	0.009	0.02

Model 4: Visual distance with demonstrator

	mean	sd	5.5%	94.5%
a[Solution]	0.667	0.022	0.632	0.701
a[Heuristic]	0.385	0.022	0.351	0.42
b[Solution]	0.991	0.002	0.988	0.994
b[Heuristic]	1.003	0.002	0.999	1.006
sigma	1.603	0.027	1.562	1.646
a[Chance]	0.269	0.008	0.256	0.282
a[Heuristic] – a[Solution]	–0.282	0.031	–0.331	–0.233
b[Heuristic] – b[Solution]	0.012	0.003	0.007	0.017

Model 5: Structural distance with demonstrator

	mean	sd	5.5%	94.5%
a[Asocial]	5156.131	43.003	5087.199	5224.872
a[Solution]	6081.134	44.372	6011.627	6150.692
a[Heuristic]	5958.768	43.377	5887.658	6028.95
sigma	391.034	3.498	385.58	396.873
a[Solution] – a[Asocial]	925.003	63.334	827.418	1023.829
a[Heuristic] – a[Asocial]	802.638	60.925	702.819	897.249
a[Heuristic] – a[Solution]	–122.366	63.229	–224.801	–22.538

Model 6: Total score

	mean	sd	5.5%	94.5%
a[Asocial]	502.174	8.997	487.379	516.011
a[Solution]	546.667	9.205	531.512	561.193
a[Heuristic]	527.149	9.056	513.029	541.491
sigma	80.855	1.489	78.377	83.306
a[Solution] – a[Asocial]	44.493	12.741	23.712	64.392
a[Heuristic] – a[Asocial]	24.975	12.65	4.501	45.456
a[Heuristic] – a[Solution]	–19.517	12.499	–39.575	0.061

Model 7: Score at the last trial

Fig. S6. Output of pre-registered statistical models

	mean	sd	5.5%	94.5%
a[Asocial]	9.133	0.253	8.715	9.522
a[Solution_2d_gen]	7.854	0.251	7.454	8.254
a[Heuristic_2d_gen]	9.159	0.255	8.741	9.564
a[Solution_3rd_gen]	7.096	0.245	6.694	7.481
a[Heuristic_3rd_gen]	8.641	0.263	8.223	9.056
sigma	2.34	0.078	2.219	2.466
a[Solution_2d_gen] – a[Asocial]	–1.278	0.338	–1.8	–0.749
a[Heuristic_2d_gen] – a[Asocial]	0.026	0.36	–0.549	0.598
a[Heuristic_2d_gen] – a[Solution_2d_gen]	1.304	0.351	0.743	1.878
a[Heuristic_3rd_gen] – a[Asocial]	–0.492	0.357	–1.056	0.081
a[Heuristic_3rd_gen] – a[Solution_2d_gen]	0.787	0.365	0.194	1.357
a[Heuristic_3rd_gen] – a[Heuristic_2d_gen]	–0.518	0.361	–1.091	0.041
a[Solution_3rd_gen] – a[Asocial]	–2.036	0.34	–2.552	–1.483
a[Solution_3rd_gen] – a[Solution_2d_gen]	–0.758	0.357	–1.317	–0.198
a[Heuristic_3rd_gen] – a[Solution_3rd_gen]	1.545	0.349	0.992	2.113
'Heuristic_3rd_gen] – a[Heuristic_2d_gen]) – (a[Solution_3rd_gen] – a[Solution_2d_gen])	0.24	0.495	–0.561	1.045

Model S1: Number of unique configurations submitted

	mean	sd	5.5%	94.5%
a[Asocial]	0.685	0.014	0.663	0.707
a[Solution_2d_gen]	0.548	0.013	0.527	0.569
a[Heuristic_2d_gen]	0.645	0.014	0.622	0.667
a[Solution_3rd_gen]	0.462	0.014	0.44	0.484
a[Heuristic_3rd_gen]	0.6	0.014	0.578	0.622
a[Solution_2d_gen] – a[Asocial]	–0.137	0.019	–0.167	–0.106
a[Heuristic_2d_gen] – a[Asocial]	–0.04	0.02	–0.073	–0.009
a[Heuristic_2d_gen] – a[Solution_2d_gen]	0.097	0.019	0.066	0.127
a[Heuristic_3rd_gen] – a[Asocial]	–0.085	0.019	–0.115	–0.053
a[Heuristic_3rd_gen] – a[Solution_2d_gen]	0.052	0.02	0.021	0.084
a[Heuristic_3rd_gen] – a[Heuristic_2d_gen]	–0.045	0.02	–0.078	–0.012
a[Solution_3rd_gen] – a[Asocial]	–0.223	0.019	–0.253	–0.194
a[Solution_3rd_gen] – a[Solution_2d_gen]	–0.086	0.019	–0.116	–0.056
a[Heuristic_3rd_gen] – a[Solution_3rd_gen]	0.139	0.019	0.109	0.17
Heuristic_3rd_gen – Solution_3rd_gen] – a[Heuristic_2d_gen – Solution_2d_gen]	0.042	0.027	–0.001	0.086

Model S2: Within-Individual visual diversity

	mean	sd	5.5%	94.5%
a[Asocial]	0.512	0.012	0.493	0.53
a[Solution_2d_gen]	0.431	0.011	0.414	0.448
a[Heuristic_2d_gen]	0.479	0.011	0.462	0.496
a[Solution_3rd_gen]	0.366	0.011	0.348	0.384
a[Heuristic_3rd_gen]	0.441	0.011	0.424	0.458
a[Solution_2d_gen] – a[Asocial]	–0.081	0.016	–0.106	–0.055
a[Heuristic_2d_gen] – a[Asocial]	–0.033	0.016	–0.059	–0.008
a[Heuristic_2d_gen] – a[Solution_2d_gen]	0.048	0.015	0.024	0.073
a[Heuristic_3rd_gen] – a[Asocial]	–0.071	0.015	–0.095	–0.046
a[Heuristic_3rd_gen] – a[Solution_2d_gen]	0.01	0.015	–0.014	0.033
a[Heuristic_3rd_gen] – a[Heuristic_2d_gen]	–0.038	0.015	–0.061	–0.014
a[Solution_3rd_gen] – a[Asocial]	–0.146	0.016	–0.171	–0.121
a[Solution_3rd_gen] – a[Solution_2d_gen]	–0.065	0.016	–0.09	–0.04
a[Heuristic_3rd_gen] – a[Solution_3rd_gen]	0.075	0.015	0.051	0.099
Heuristic_3rd_gen – Solution_3rd_gen] – a[Heuristic_2d_gen – Solution_2d_gen]	0.027	0.021	–0.008	0.061

Model S3: Within-Individual structural diversity

	mean	sd	5.5%	94.5%
a[Solution_2d_gen]	0.48	0.02	0.448	0.513
a[Heuristic_2d_gen]	0.139	0.021	0.103	0.172
a[Solution_3rd_gen]	0.313	0.023	0.276	0.348
a[Heuristic_3rd_gen]	0.135	0.021	0.1	0.168
Chance	0.13	0.006	0.119	0.14
a[Heuristic_2d_gen] – a[Solution_2d_gen]	–0.341	0.029	–0.39	–0.295
a[Solution_3rd_gen] – a[Solution_2d_gen]	–0.166	0.031	–0.216	–0.118
a[Heuristic_3rd_gen] – a[Solution_2d_gen]	–0.345	0.03	–0.394	–0.298
a[Heuristic_3rd_gen] – a[Heuristic_2d_gen]	–0.004	0.03	–0.053	0.045
a[Heuristic_3rd_gen – Solution_3rd_gen]	–0.178	0.031	–0.227	–0.13
Heuristic_3rd_gen – Solution_3rd_gen] – a[Heuristic_2d_gen – Solution_2d_gen]	0.162	0.043	0.094	0.23

Model S4: Visual distance with demonstrator

	mean	sd	5.5%	94.5%
a[Solution_2d_gen]	0.613	0.02	0.581	0.645
a[Heuristic_2d_gen]	0.399	0.019	0.37	0.431
a[Solution_3rd_gen]	0.474	0.02	0.442	0.506
a[Heuristic_3rd_gen]	0.361	0.019	0.33	0.391
Chance	0.269	0.008	0.256	0.282
a[Heuristic_2d_gen] – a[Solution_2d_gen]	–0.214	0.027	–0.256	–0.171
a[Solution_3rd_gen] – a[Solution_2d_gen]	–0.139	0.028	–0.183	–0.096
a[Heuristic_3rd_gen] – a[Solution_2d_gen]	–0.252	0.027	–0.294	–0.212
a[Heuristic_3rd_gen] – a[Heuristic_2d_gen]	–0.038	0.026	–0.08	0.003
a[Heuristic_3rd_gen – Solution_3rd_gen]	–0.112	0.027	–0.156	–0.069
Heuristic_3rd_gen – Solution_3rd_gen] – a[Heuristic_2d_gen – Solution_2d_gen]	0.101	0.038	0.04	0.163

Model S5: Structural distance with demonstrator

	mean	sd	5.5%	94.5%
a[Asocial]	5156.23	53.568	5071.916	5241.547
a[Solution_2d_gen]	6080.028	50.921	5998.726	6162.312
a[Heuristic_2d_gen]	5959.703	50.861	5876.025	6043.261
a[Solution_3rd_gen]	6494.217	49.961	6417.585	6575.126
a[Heuristic_3rd_gen]	6376.099	50.79	6298.032	6457.435
sigma	462.419	3.78	456.167	468.58
a[Solution_2d_gen] – a[Asocial]	923.798	74.29	805.618	1044.931
a[Heuristic_2d_gen] – a[Asocial]	803.474	72.459	682.96	920.725
a[Heuristic_2d_gen] – a[Solution_2d_gen]	–120.324	72.221	–236.987	–9.187
a[Heuristic_3rd_gen] – a[Asocial]	1219.87	76.882	1098.098	1344.109
a[Heuristic_3rd_gen] – a[Solution_2d_gen]	296.072	71.697	180.301	410.617
a[Heuristic_3rd_gen] – a[Heuristic_2d_gen]	416.396	71.628	302.843	534.324
a[Solution_3rd_gen] – a[Asocial]	1337.988	73.959	1218.541	1456.207
a[Solution_3rd_gen] – a[Solution_2d_gen]	414.19	70.524	308.48	526.831
a[Heuristic_3rd_gen] – a[Solution_3rd_gen]	–118.118	69.315	–227.317	–6.597
Heuristic_3rd_gen – Solution_3rd_gen] – a[Heuristic_2d_gen – Solution_2d_gen]	2.206	99.78	–154.416	163.965

Model S6: Total score

	mean	sd	5.5%	94.5%
a[Asocial]	502.492	10.427	485.308	518.858
a[Solution_2d_gen]	546.27	10.613	529.666	563.372
a[Heuristic_2d_gen]	526.521	10.214	509.691	542.744
a[Solution_3rd_gen]	558.383	10.4	541.344	574.389
a[Heuristic_3rd_gen]	544.924	10.2	528.85	561.358
sigma	93.032	1.625	90.351	95.617
a[Solution_2d_gen] – a[Asocial]	43.778	14.944	20.154	68.831
a[Heuristic_2d_gen] – a[Asocial]	24.029	14.396	1.2	47.111
a[Heuristic_2d_gen] – a[Solution_2d_gen]	–19.749	14.772	–44.023	3.967
a[Heuristic_3rd_gen] – a[Asocial]	42.432	15.238	17.351	66.884
a[Heuristic_3rd_gen] – a[Solution_2d_gen]	–1.346	14.684	–24.548	22.409
a[Heuristic_3rd_gen] – a[Heuristic_2d_gen]	18.403	14.571	–4.904	40.515
a[Solution_3rd_gen] – a[Asocial]	55.891	14.279	32.475	78.831
a[Solution_3rd_gen] – a[Solution_2d_gen]	12.113	15.167	–12.584	35.016
a[Heuristic_3rd_gen] – a[Solution_3rd_gen]	–13.459	14.832	–37.171	9.784
Heuristic_3rd_gen – Solution_3rd_gen] – a[Heuristic_2d_gen – Solution_2d_gen]	6.29	21.386	–28.078	39.685

Model S7: Score at the last trial

Fig. S7. Output of exploratory statistical models including third generation participants

	mean	sd	5.5%	94.5%
a[Asocial]	0.451	0.054	0.365	0.538
a[Solution_2d_gen]	0.464	0.054	0.377	0.552
a[Heuristic_2d_gen]	0.537	0.056	0.446	0.625
a[Solution_3rd_gen]	0.41	0.057	0.32	0.5
a[Heuristic_3rd_gen]	0.608	0.053	0.52	0.694
a[Solution_2d_gen] – a[Asocial]	0.012	0.078	–0.113	0.139
a[Heuristic_2d_gen] – a[Asocial]	0.085	0.078	–0.043	0.209
a[Solution_3rd_gen] – a[Asocial]	–0.042	0.078	–0.162	0.085
a[Heuristic_3rd_gen] – a[Asocial]	0.157	0.075	0.038	0.273
a[Heuristic_2d_gen] – a[Solution_2d_gen]	0.073	0.081	–0.061	0.203
a[Solution_3rd_gen] – a[Solution_2d_gen]	–0.054	0.079	–0.18	0.079
a[Heuristic_3rd_gen] – a[Solution_2d_gen]	0.144	0.075	0.025	0.266
a[Solution_3rd_gen] – a[Heuristic_2d_gen]	–0.127	0.081	–0.254	0.004
a[Heuristic_3rd_gen] – a[Heuristic_2d_gen]	0.071	0.076	–0.047	0.191
a[Heuristic_3rd_gen] – a[Solution_3rd_gen]	0.198	0.08	0.066	0.33

Fig. S8. Output of exploratory statistical models on the proportion of correct heuristics
